# Genome Sequence of Subcluster L3 Mycobacterium smegmatis mc^2^155 Phage DuncansLeg

**DOI:** 10.1128/mra.01069-22

**Published:** 2022-12-05

**Authors:** Megan Cevasco, Kaitlynn Burbage, Connor Hadwin, Edenborough Hibionada, Kirsten Presnell, Daniel Williams

**Affiliations:** a Department of Biology, Coastal Carolina University, Conway, South Carolina, USA; DOE Joint Genome Institute

## Abstract

Bacteriophage DuncansLeg is a siphovirius isolated in 2021 from soil on the Coastal Carolina University campus in Conway, South Carolina, using the host Mycobacterium smegmatis mc^2^155. DuncansLeg has a 75,593-bp circular genome that contains 126 predicted protein-coding genes and 10 tRNA genes. DuncansLeg is assigned to actinobacteriophage cluster L3.

## ANNOUNCEMENT

**FIG 1:**
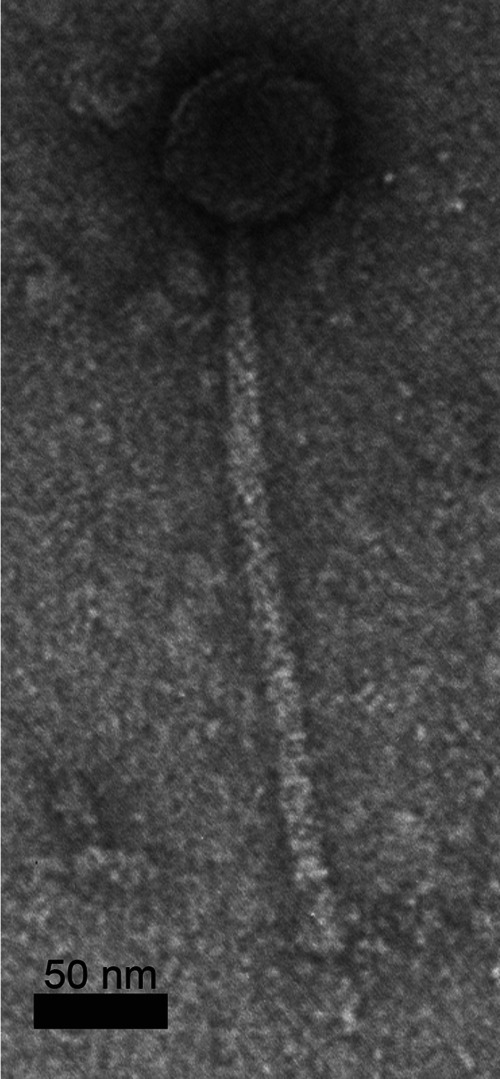
Transmission electron micrograph (JEOL JEM 1230, imaged at 100 kV) of bacteriophage DuncansLeg negatively stained (1% uranyl acetate).

Bacteriophages that target *Actinobacteria* within the genus Mycobacterium are of biomedical interest as potential therapeutics for treating patients infected with mycobacterial pathogens, such as Mycobacterium abscessus (1). Here, we present the genome sequence of a novel mycobacteriophage, DuncansLeg, isolated using nonpathogenic Mycobacterium smegmatis mc^2^155. DuncansLeg was isolated in October 2021 from a loamy moist soil sample which included some plant roots and was collected from Coastal Carolina University’s campus in Conway, South Carolina (global positioning system [GPS] 33.7993N, 79.0138W). Using standard protocols (2), the soil sample was washed with 7H9 liquid medium, and the wash was filtered using a 0.22-μm syringe filter. The filtrate was then inoculated with Mycobacterium smegmatis and incubated with shaking for approximately 48 h at 37°C before being filtered and plated onto 7H9 top agar containing Mycobacterium smegmatis. DuncansLeg produced clear plaques with haloed margins ~1.5 mm in diameter after 2 days at 37°C. DuncansLeg was purified through two rounds of plating. Transmission electron microscopy of DuncansLeg (Fig. 1) displayed a siphovirus morphotype with an icosahedrally symmetric capsid with a mean diameter of 63.9 nm (SD, 3.3) and a noncontractile tail with a mean length of 277.8 nm (SD, 14.1) (*n* = 9) as measured using ImageJ software (https://imagej.nih.gov/ij).

Double-stranded DNA was isolated from the high-titer lysate (>5 × 10^9^ PFU/mL) of DuncansLeg using the Promega Wizard DNA cleanup kit and prepared for sequencing on an Illumina MiSeq platform (v3 reagents) using the New England BioLabs (NEB) Ultra II library kit. This sequencing protocol resulted in 361,011 single-end 150-bp reads across the DuncansLeg genome, providing 677-fold coverage. Untrimmed reads were assembled using Newbler v2.9 and then were checked for completeness with Consed v29 following the methods outlined in Russell (3). DuncansLeg has a genome of 75,593 bp with a G+C content of 59.3% and 3′ single-stranded genome ends (5′-TCGATCAGCC-3′). Based upon a gene content similarity of 35% or higher to phages in the Actinobacteriophage database (http://phagesDB.org) and using previously described criteria, DuncansLeg is assigned to subcluster L3 (4, 5).

The genome was autoannotated using GLIMMER v3.02 (6) and GeneMark v2.5 (7) embedded in DNAMaster v5.23.2 (http://cobamide2.bio.pitt.edu/computer.htm) and then manually refined using PECAAN (http://discover.kbrinsgd.org) and Starterator v1.0.1 (https://seaphages.org/software/#Starterator). In total, 136 protein-coding genes were predicted for DuncansLeg, of which functions could be assigned to 50 predicted genes using Phamerator v472 (8), BLASTP (nonredundant protein sequence database) v2.13 (9), and HHpred (protein data bank database) (10). ARAGORN v1.2.38 (11) and tRNAscan-SE v2.0 (12) were used to identify 10 tRNA genes. All software was used with default settings.

The first 30 predicted genes in the genome include structure and assembly genes, as well as lysin A, lysin B, and holin, of which all are transcribed on one strand. The remaining genome is occupied by DNA metabolism and tRNA genes, as well as many predicted genes of unknown function, where a minority of these genes are transcribed on the opposite or other strand. DuncansLeg encodes a predicted HicA-like toxin antitoxin system, numerous nucleases and DNA-binding proteins, RNA ligase, and a ClpP protease. DuncansLeg also encodes a predicted immunity repressor, tyrosine integrase, and excise and is therefore likely a temperate phage, which is consistent with other phages in cluster L.

### Data availability.

DuncansLeg is available at GenBank with accession no. ON755181.1 and Sequence Read Archive (SRA) no. SRX14443497.
